# Serum programmed death ligand 2 is elevated in cats with mammary carcinoma

**DOI:** 10.1038/s41598-026-41375-9

**Published:** 2026-02-24

**Authors:** Vitória Silva João, Gonçalo Pereira, Gonçalo Vicente, Ana Catarina Urbano, Jorge Correia, João Ferreira, Fernando Ferreira

**Affiliations:** 1 Centre for Interdisciplinary Research in Animal Health (CIISA), Faculty of Veterinary Medicine, University of Lisbon, Lisbon, 1300-477 Portugal; 2 Associate Laboratory for Animal and Veterinary Sciences (AL4AnimalS), Lisbon, 1300-477 Portugal; 3 Veterinary Teaching Hospital, Faculty of Veterinary Medicine, University of Lisbon, Lisbon, 1300-477 Portugal; 4 Dermatology Research Unit, Faculty of Medicine, University of Lisbon, Lisbon, Portugal

**Keywords:** Feline mammary carcinoma, PD-L2, Biomarker, ELISA, Comparative oncology model, Biomarkers, Cancer, Oncology

## Abstract

Since the PD-1/PD-L1/PD-L2 axis plays a vital role in immune tolerance and T-cell exhaustion, having emerged as a target for breast cancer immunotherapy, we investigated the relevance of serum PD-L2 (sPD-L2) levels in feline mammary carcinoma (FMC), to validate its potential use as a diagnostic and/or prognostic biomarker, and as a future target for immunotherapy. To accomplish that, sPD-L2 levels were quantified by enzyme-linked immunosorbent assay and compared between healthy control cats and cats with mammary carcinoma, also stratified by tumor molecular subtype. Statistical associations between sPD-L2 levels, clinicopathological features and other serum immune checkpoint molecules (sPD-1, sPD-L1, sCTLA-4, sTNF-α, sVEGF-A, sVEGFR-1, sVEGFR-2 and sLAG-3) were also analyzed. Results revealed that sPD-L2 levels were significantly higher in the FMC group (*p* < 0.0001), with a concentration of 1934 pg/mL established as the best cut-off value to distinguish sick from healthy cats (specificity: 96.6%; sensitivity: 93.6%; AUC = 0.980). Interestingly, cats with HER2-positive or Triple-Negative (TN) mammary carcinoma subtypes showed higher sPD-L2 levels (*p* < 0.0001). According to receiver-operating characteristic (ROC) curve analysis, a serum PD-L2 concentration of 5499 pg/mL represented the optimal cut-off for distinguishing cats with these two subtypes from cats with Luminal A (LA) and Luminal B (LB) carcinomas (specificity: 95.2%; sensitivity: 82.6%; AUC = 0.919). Furthermore, in the FMC group, positive correlations were found between sPD-L2 levels and sCTLA-4 (*r* = 0.496, *p* = 0.001), sTNF-α (*r* = 0.482, *p* = 0.0009), sVEGF-A (*r* = 0.54, *p* = 0.0002), sVEGFR-1 (*r* = 0.339, *p* = 0.025), sVEGFR-2 (*r* = 0.322, *p* = 0.033) and sLAG-3 levels (*r* = 0.324, *p* = 0.032). Finally, significant associations were found between sPD-L2 levels and progesterone receptor (PR) status (*p* = 0.002), HER2 status (*p* = 0.009) and Ki-67 index (*p* < 0.0001). A serum sPD-L2 concentration of 3732 pg/mL was identified as the optimal cut-off value for distinguishing FMCs with a high Ki-67 index (≥ 14%) from those with a low index (specificity: 80.0%; sensitivity: 94.1%; AUC = 0.906). In conclusion, our findings suggest that sPD-L2 is a potential biomarker for FMC, particularly in HER2-positive and TN subtypes.

## Introduction

Immune checkpoints play a pivotal role in regulation of immune homeostasis, becoming critical targets in cancer immunotherapy, where they modulate immune escape and antitumor immunity^1^. Importantly, checkpoint molecules can exist in distinct molecular forms and cellular compartments, that can show divergent biological functions, clinical associations and therapeutic implications. For ligands (e.g. PD-L1, PD-L2), membrane-bound expression reflects a spatially restricted, cell–cell regulatory axis operating at the tumor–immune interface, whereas soluble counterparts represent a systemically measurable pool that may arise through proteolytic shedding, alternative splicing or release on extracellular vesicles. These forms can therefore capture different aspects of tumor biology and host response, and they should be discussed as related but non-equivalent entities. Indeed, the membrane-bound checkpoint proteins (e.g., cell-surface PD-L1 assessed by immunohistochemistry or flow cytometry) have been extensively studied because they directly mediate inhibitory signaling in situ by engaging receptors such as PD-1 on T cells, thereby attenuating proliferation, cytokine production and cytotoxic function within the tumor microenvironment^1–3^. As a consequence, cell membrane expression has often been used as a benchmark readout in translational oncology and in patient stratification for checkpoint blockade. In this context, localization matters: expression on tumor cells, antigen-presenting cells, or stromal/immune subsets may carry distinct mechanistic interpretations and may correlate differently with clinicopathologic variables and treatment response. By contrast, soluble checkpoint proteins (e.g., serum sPD-L1 levels) can retain receptor-binding capacity and modulate signaling at a distance, potentially acting as decoys or competitors that alter the availability of PD-1/PD-L interactions, or as systemic immunomodulators that reflect broader inflammatory or immunoregulatory states^1,3^. Depending on their origin and molecular composition, soluble forms may also mirror tumor burden, immune-cell activation, protease activity, or microenvironmental remodeling. Consequently, associations between circulating levels and clinical outcomes may be robust and clinically useful, yet they do not necessarily imply the same causal pathway as membrane expression within tumor tissue.

These different molecular forms and cellular localizations have direct prognostic and therapeutic implications. While the membrane expression is conceptually linked to local immune suppression and therefore to the mechanistic target of many checkpoint inhibitors, the soluble forms may integrate multiple biological processes and can change dynamically with systemic inflammation, comorbid conditions, or treatment-induced immune modulation. A biomarker based on soluble proteins may offer practical advantages (minimally invasive sampling, longitudinal monitoring), but its interpretation requires explicit acknowledgement that it is not a surrogate measure of surface expression unless concordance is empirically demonstrated. In light of these considerations, studies evaluating soluble checkpoint molecules should explicitly frame them as circulating immunoregulatory biomarkers with potentially independent clinical meaning from tissue expression. Where feasible, parallel assessment of membrane-bound expression in tumor and immune compartments, together with correlation analyses between tissue and serum measures, can clarify whether soluble levels track local checkpoint biology or reflect broader systemic processes. Such a structured distinction strengthens mechanistic interpretation, improves comparability across studies, and prevents overextension of conclusions derived from one molecular form to another.

Considering the PD-1/PD-L1/PD-L2 axis, the interaction between the PD-1 receptor and its ligands leads to T cell-mediated immunity suppression, reducing antigen-specific responses, like T cell proliferation, cytokine secretion and cytotoxicity^4,5^. While PD-L1 expression is present in both hematopoietic and non-hematopoietic cells, PD-L2 expression is mainly limited to antigen-presenting cells^4^, although both ligands can be expressed by tumor and stromal cells^6^. Despite being part of the B7 family and sharing 40% of amino acid identity^7^, the binding affinity of PD-L2 to PD-1 is 2 to 6 times higher than that of PD-L1^8^, suggesting that PD-L2 may play a greater role in modulating the immune response of tumor infiltrating lymphocytes (TILs)^9^. In cancer cells, PD-L2 expression has been associated with anti-PD-L1 immunotherapy resistance in preclinical animal models and in humans^10^, supporting its role in immune evasion^11^. Thus, PD-L2 has been suggested as a potential biomarker and target for immunotherapy^12^, but the literature remains limited. Notably, although anti-PD-1/PD-L1 antibodies can reactivate T cell effector functions^5^ with a promising and effective treatment response in advanced cancer^13,14^, many patients have shown an incomplete clinical response and/or therapeutic resistance, potentially due to tumoral PD-L2 expression^15^.

Indeed, the selection of candidates for PD-1/PD-L1 immune checkpoint blockade therapy typically disregards PD-L2 expression^16^, overlooking its potential for stratifying patients with more aggressive tumors who might benefit from other immunotherapies^17^.

Clinically, multiple studies and systematic reviews indicate that elevated sPD-L1 is frequently associated with adverse clinicopathological features and poorer survival in several solid tumors, supporting its role as a prognostic correlate rather than a stand-alone diagnostic marker^18^.

In the immunotherapy setting, sPD-L1 has been explored as a pharmacodynamic biomarker, where baseline levels and on-treatment changes may relate to outcomes, yet require assay standardization and prospective validation^19^.

Although PD-L2 is less characterized than sPD-L1, several reports have shown that different cancers overexpress PD-L2, including ovarian cancer^17^, lung adenocarcinoma^20,21^, lung squamous cell carcinoma^22^, renal cell carcinoma^23^, pancreatic ductal adenocarcinoma^24^, gastric cancer^25^, esophageal squamous cell carcinoma^26^, head and neck squamous cell carcinoma^12^, and breast cancer^15,27–32^.Furthermore, PD-L2 overexpression is associated with poorer prognosis^33^ and positively correlated with elevated PD-L1 levels in different cancer types, including breast cancer^15^.

Nowadays, breast cancer is the most frequently diagnosed cancer in humans^34^, and feline mammary carcinoma represents a relevant biological model as both share many clinicopathological features^35,36^. Notably, the IHC-based breast cancer molecular classification proposed by the St. Gallen International Expert Consensus^37^ was successfully applied in FMC^36^, since in both species, mammary carcinomas can be classified as Luminal A (LA), Luminal B (LB), HER2-positive and Triple Negative (TN) (normal like and basal-like)^38,39^. This molecular classification is based on the expression of estrogen receptor (ER), progesterone receptor (PR), human epidermal growth factor receptor-type 2 (HER2), cytokeratin 5/6 (CK5/6) and Ki-67^40^. Thus, while LA tumors demonstrate an overexpression of ER and/or PR receptors, lack of HER2 expression and low Ki-67 index levels, being associated with better prognosis, LB carcinomas are more aggressive than LA tumors and are commonly subdivided into LB/HER2-negative (ER and/or PR positive, HER2 negative, high Ki-67) and LB/HER2-positive (ER and/or PR positive with HER2 positivity)^37^. In contrast, HER2-enriched and triple-negative (TN) subtypes are typically more aggressive and associated with poorer outcomes than LA and LB tumors^41^. HER2-positive carcinomas show HER2 overexpression with absent or minimal expression of hormone receptors^40^. Finally, TN tumors lack ER, PR, and HER2 expression and generally carry the worst prognosis. Within TN disease, basal-like and normal-like phenotypes can be distinguished by cytokeratin expression, with basal-like tumors expressing CK5/6, CK14, and CK17^42^.

As in human breast cancer (HBC) patients, cats with mammary carcinoma show higher serum PD-1 and PD-L1 levels than healthy controls, particularly cats with HER2-positive and Triple-Negative (TN) tumor subtypes^43^. However, no studies have yet addressed the role of serum PD-L2 levels in cats, despite the extensive sequence homology between feline and human PD-L2 (~ 81%)^44^, suggesting that its biological role might be conserved.

Thus, in this study we: (i) compared the serum PD-L2 (sPD-L2) levels between cats with mammary carcinoma and healthy controls; (ii) evaluated the sPD-L2 levels in cats stratified by tumor molecular subtype (Luminal A, Luminal B, HER2-positive, Triple-negative); (iii) assessed significant associations between sPD-L2 levels and various clinicopathological features (breed, age, reproductive status, tumor size and localization, lymph node stage, tumor ulceration and necrosis, treatment, presence of multiple tumors, stage TNM, disease recurrence, Histopathologic classification, malignancy grade, lymphatic invasion, lymphatic infiltration, PR status, ER status, HER2 status, CK5/6 status, molecular classification), serum immune checkpoint molecule levels (sPD-1, sPD-L1, sCTLA-4, sTNF-α, sVEGF-A, sVEGFR-1, sVEGFR-2 and sLAG-3) and the mitotic Ki-67 index; (iv) investigate the potential use of sPD-L2 as a prognostic biomarker.

## Materials and methods

### Animal population

Blood samples were collected from 28 healthy queens (asymptomatic animals with normal blood analyses, negative for FIV/FeLV, and undergoing elective ovariohysterectomy at the Veterinary Teaching Hospital of the Faculty of Veterinary Medicine, University of Lisbon) and 52 queens diagnosed with mammary carcinoma prior to unilateral mastectomy and ovariectomy. Approximately 1 mL of peripheral blood was collected into a dry tube with gel from femoral or jugular veins, and then centrifugated for 5 min at 4900 rpm. Subsequently, the serum was transferred to microtubes and stored at -20 °C until further use. All hemolyzed samples were discarded, as recommended for ELISA procedures^45^. The use of animal-derived samples in this study was used in accordance with ARRIVE guidelines (https://arriveguidelines.org), and approved by the Teach and Research Ethics Committee (CEIE, Comissão de Ética para a Investigação e Ensino) from the Faculty of Veterinary Medicine, University of Lisbon, under approval number 010/2025. Furthermore, all methods were performed in accordance with the relevant guidelines and regulations (Portuguese Decree–Law No. 113/2013 of August 7, 2013, which implements Directive 2010/63/EU of the European Parliament and Council).

In parallel, the clinicopathological features of the cats enrolled in this study were recorded including age, breed, reproductive status, contraceptive use (megestrol acetate), treatment performed (none, surgery or surgery plus doxorubicin-based chemotherapy after definitive surgery in animals with high-risk features as large primary tumor, lymph node involvement, high-grade histology, lymphovascular invasion and metastatic disease), number, location, and size of tumor lesions, as previously reported^43^; histopathological classification^46,47^ and malignancy grade^48,49^; presence of metastasis (assessed by CT scan/thoracic radiographs combined with abdominal ultrasonography), tumor necrosis, lymphatic invasion, lymphocytic infiltration, cutaneous ulceration, regional lymph node involvement (assessed by surgical excision and subsequent histopathological examination of the axillary lymph node for the cranial mammary glands [M1–M3] and the superficial inguinal lymph node for the caudal mammary glands [M4–M5]); stage of the disease according to the TNM system^50^; molecular classification^36^ and the Ki-67 index using a cut-off value previously reported^51^.

The clinical staging was performed by using the TNM system, which describes the anatomic extent of disease through three components: T (primary tumor), N (regional lymph nodes), and M (distant metastasis). The T category is primarily based on the largest diameter of the mammary mass and local invasion of adjacent tissues. The N category reflects whether regional lymph nodes draining the mammary chain are involved by metastasis, confirmed by cytology or histopathology. The M category indicates the presence (M1) or absence (M0) of distant metastasis, using thoracic imaging for pulmonary involvement and abdominal imaging. These TNM components were then combined into stage groups that provide a clinically intuitive stratification of prognosis and guide therapeutic planning. The stage I corresponds to a small, localized primary tumor (low T) with no evidence of regional nodal spread (N0) and no distant metastasis (M0). Stage II denotes a larger primary tumor and/or early regional nodal involvement while still lacking distant metastasis (i.e., M0), reflecting a higher local-regional disease burden than stage I. Stage III represents locally advanced disease, characterized by a large and/or invasive primary tumor and/or more extensive regional lymph node metastasis, again without distant metastasis (M0). Finally, stage IV denotes metastatic disease and is defined by the presence of distant metastasis (M1) regardless of primary tumor size or nodal status.

Tumor necrosis was assessed histopathologically on H&E-stained sections, defined as the presence of intratumoral areas of cell death characterized by loss of tissue architecture, eosinophilic/granular necrotic material, cellular debris with nuclear karyorrhexis/karyolysis and, in some cases, ghost cells, often with a peripheral inflammatory reaction.

The molecular classification of the tumors was based on previously published immunohistochemical results^36^.

Overall survival and disease-free survival were determined for each animal through the medical records from follow-up appointments.

### Serological quantification of PD-L2

Serum PD-L2 levels were measured using a solid-phase sandwich ELISA (PD-L2/B7-DC DuoSet ELISA, #DY1224, R&D Systems, Inc, Minneapolis, USA) following the manufacturer’s recommendations. Although this ELISA kit has been extensively validated for use with human serum samples^52–54^, its use in feline serum samples was undertaken for the first time in this study, since serum PD-L2 levels were never analyzed in cats.

A standard curve was generated using serial dilutions of recombinant PD-L2 (625, 1,250, 2,500, 5,000, 10,000, 20,000, 40,000 and 80,000 pg/mL), and concentrations were calculated by linear regression (0,0005x − 0,0623, R^2^ = 0,9997for rPD-L2) considering the distribution of the readouts and the manufacturer’s instructions.

Briefly, 96-well microplates were coated overnight at room temperature (RT) with 100 µL/well of the capture antibody. The following day, wells were washed three times with 400 µL of 0.05% PBS-Tween (PBS-T) and blocked with 300 µL/well of 1% (w/v) BSA in PBS for 60 min at RT. After three additional washes with PBS-T, 100 µL/well of standard dilutions were added in duplicate. Serum samples were diluted 1:20 in 1% BSA in PBS, and 100 µL of each serum sample was added to the wells. Then, plates were covered and incubated for 2 h at RT, followed by three washes with PBS-T.

Next, 100 µL/well of detection antibody was added and incubated for 2 h at RT. After three additional washes, 100 µL/well of streptavidin-HRP was added and incubated for 25 min at RT, protected from light. Plates were washed (3 × 400 µL/well PBS-Tween 0.05%), 100 µL/well of substrate solution (1:1 mixture of H2O2 and tetramethylbenzidine) was added and incubated for 20 min at RT in the dark. Finally, the reaction was stopped by adding 50 µL/well of 2 N H2SO4. The optical density of each well was determined within 5 min using a microplate reader set to 450 nm (Fluostar Optima Microplate Reader, BMG, Ortenberg, Germany). To correct optical imperfections in the plate, a second reading was performed at 540–570 nm. Then readings obtained at these wavelengths were subtracted from readings obtained at 450 nm.

In parallel, correlations between the serum PD-L2 concentrations determined in this study and the previously reported sPD-1/PD-L1, sCTLA-4, sTNF-α^43^, sVEGF-A, sVEGFR-1, sVEGFR-2 and sLAG-3 levels^55^ were investigated applying the Spearman’s rank correlation coefficient.

### Statistical analysis

The Mann-Whitney U test was used to compare sPD-L2 levels between cats with mammary carcinoma and healthy controls, as well as to assess associations between sPD-L2 levels and hormonal receptor status (PR and ER), reproductive status, contraceptive use, presence of metastasis, tumor necrosis, lymphatic invasion, lymphocytic infiltration, skin ulceration, involvement of regional lymph nodes, Ki-67 index, CK5/6 and HER2 status.

The Kruskal–Wallis’s test followed by Dunn’s multiple comparisons test was used to assess associations between sPD-L2 levels and molecular FMC subtypes (Luminal A, Luminal B, HER2-positive and TN), as well as TNM stage and various clinicopathological parameters, including age, breed, treatment, tumor characteristics (number, location and size), histopathological classification, and malignancy grade.

Optimal cut-off values were determined using the Youden index, and Receiver Operating Characteristic (ROC) curves were used to represent the performance of sPD-L2 in distinguishing diseased cats from healthy controls, differentiating aggressive (HER2-positive and TN subtypes) from less aggressive (Luminal A and Luminal B) subtypes, and discriminating between cats with high (≥ 14%) and low (< 14%) Ki-67 indices.

Kaplan-Meier curves were used to graphically represent overall survival (OS) and disease-free survival (DFS), according to FMC molecular subtype and sPD-L2 levels, with animals stratified into high (≥ median) and low (< median) sPD-L2 groups. The time between the initial diagnosis and the death of the animal related to the disease represents OS, whereas the DFS is defined as the time from surgery to relapse (local recurrence, regional/distant metastasis) or death from cancer related causes. In this context, the OS was assessed for 30 months and the DFS was assessed for 25 months. Animals were censored if they were alive and/or disease-free at the last follow-up, lost to follow-up, or if death occurred due to causes unrelated to cancer. Loss to follow-up included failure to attend scheduled recheck appointments, inability to contact the owner by telephone, lack of communication of death to the attending veterinarian, or incomplete clinical records.

Statistical analysis was performed in GraphPad Prism version 8.1.2 (GraphPad Software, CA, USA). A two-sided p-value < 0.05 was considered statistically significant. Results are presented as median values. Graphs were generated using GraphPad Prism and Microsoft Excel for Windows (version 16.30, Microsoft Corporation, Redmond, WA, USA).

## Results

### Study population

The study population with mammary carcinoma consisted predominantly of European Shorthair cats, aged 10 years or older. Regarding the reproductive status, nearly half of the cats were spayed and had a history of contraceptive use; most underwent mastectomy, and at the end of the study most were alive (Table 1).

Multiple tumors were observed in the majority of cases, with no lymph node involvement. Additionally, TNM stage III was the most frequent, while most tumors were non-ulcerated, and the abdominal mammary glands were the most commonly affected (Table 2).

Regarding, the tumor pathological characteristics, most tumors were classified as tubulopapillary carcinomas, showing a malignancy grade of III. Necrosis was present in 41 cases and lymphocytic infiltration was observed in 31 cats, while lymphatic invasion was absent in the majority. Tumor size was most commonly 2–3 cm, and 44 cats presented with the disease for the first time (Table 3). Regarding molecular markers, 48.1% of tumors were PR-positive, 51.9% were CK5/6-positive, and 71.1% showed a Ki-67 index ≥ 14%. ER-positive and HER2-positive tumors were less frequent. Stratifying tumors by molecular subtype, 7 were Luminal A, 17 were Luminal B, 8 were Luminal B HER2-positive, 3 HER2-positive and 13 Triple-Negative, further divided into basal-like and normal-like (Table 3).

**Table 1:** Clinical features of 52 female cats with mammary carcinoma.

Clinical feature	*n* (%)	Clinical feature	*n* (%)
*n*		*n*	
European Shorthair	38 (73.1%)	Yes	23 (44.2%)
Siamese	5 (9.6%)	No	22 (42.3%)
Persian	5 (9.6%)	Unknown	7 (13.5%)
Norwegian Forest Cat	3 (5.8%)	Treatment (*n* = 52)	
Russian Blue	1 (1.9%)	None	2 (3.8%)
Age (*n* = 52)		Mastectomy	44 (84.7%)
< 10 years	3 (5.8%)	Mastectomy+ Chemotherapy	6 (11.5%)
10–14 years	42 (80.8%)	Recurrence (*n* = 52)	
> 14 years	7 (13.4%)	Yes	7 (13.5%)
Spayed (*n* = 52)		No	44 (84.6%)
Yes	27 (51.9%)	Unknown	1 (1.9%)
No	24 (46.2%)	Alive (*n* = 52)	
Unknown	1 (1.9%)	Yes	28 (53.9%)
		No	22 (42.3%)
		Unknown	2 (3.8%)

**Table 2:** Tumor clinical features.

Tumor feature	*n* (%)	Tumor feature	*n* (%)
Multiple tumors (*n* = 52)		Localization (*n* = 52)	
Yes	31 (59.6%)	M1	9 (17.3%)
No	21 (40.4%)	M2	9 (17.3%)
Lymph node stage (*n* = 52)		M3	17 (32.7%)
Positive	19 (36.5%)	M4	17 (32.7%)
Negative	30 (57.7%)	Ulceration (*n* = 52)	
Unknown	3 (5.8%)	Yes	9 (17.3%)
Stage TNM (*n* = 52)		No	43 (82.7%)
I	11 (21.2%)	Size (*n* = 52)	
II	7 (13.5%)	< 2 cm	16 (30.8%)
III	27 (51.9%)	2–3 cm	19 (36.5%)
IV	7 (13.4%)	> 3 cm	17 (32.7%)

**Table 3:** Tumor pathological features.

Pathological feature	*n* (%)	Pathological feature	*n* (%)
HP Classification (*n* = 52)		PR status (*n* = 52)	
Tubulopapillary carcinoma	21 (40.4%)	Positive	25 (48.1%)
Solid carcinoma	6 (11.5%)	Negative	23 (44.2%)
Cribriform carcinoma	3 (5.8%)	Unknown	4 (7.7%)
Mucinous carcinoma	6 (11.5%)	ER status (*n* = 52)	
Adenosquamous carcinoma	1 (1.9%)	Positive	12 (23.1%)
Tubular carcinoma	11 (21.2%)	Negative	36 (69.2%)
Papillary-cystic carcinoma	3 (5.8%)	Unknown	4 (7.7%)
Comedonic carcinoma	1 (1.9%)	HER2 status (*n* = 52)	
Malignancy Grade (*n* = 52)		Positive	11 (21.2%)
I	3 (5.8%)	Negative	37 (71.1%)
II	7 (13.4%)	Unknown	4 (7.7%)
III	42 (80.8%)	Triple-Negative (TN) (*n* = 52)	13 (25%)
Necrosis (*n* = 52)		Unknown	4 (7.7%)
Yes	41 (78.8%)	CK5/6 (1%) (*n* = 52)	
No	11 (21.2%)	Positive	27 (51.9%)
Lymphatic invasion (*n* = 52)		Negative	21 (40.4%)
Yes	10 (19.2%)	Unknown	4 (7.7%)
No	42 (80.8%)	Molecular tumor subtype classification (*n* = 52)	
Lymphatic infiltration (*n* = 52)		LA	7 (13.4%)
Yes	31 (59.6%)	LB	17 (32.7%)
No	15 (28.9%)	LB HER2	8 (15.4%)
Unknown	6 (11.5%)	HER2	3 (5.8%)
Ki67 index (*n* = 52)		TN basal-like	8 (15.4%)
Low (< 14%)	11 (21.2%)	TN normal-like	5 (9.6%)
High (> 14%)	37 (71.1%)	Unknown	4 (7.7%)
Unknown	4 (7.7%)		

HP= histopathological.

### Cats with mammary carcinoma showed higher serum PD-L2 levels than healthy controls

**Fig. 1:**
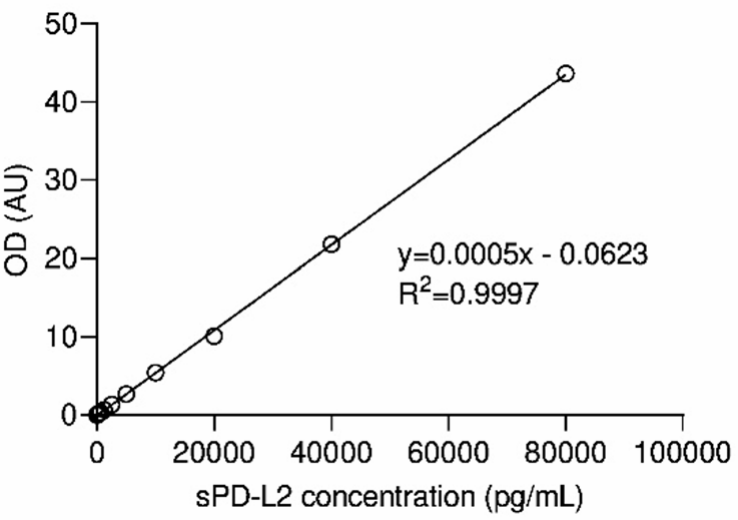
Standard curve for quantification of serum PD-L2 levels by ELISA. The standard curve was calculated plotting the mean absorbance for each standard on the y-axis against the concentration on the x-axis. R-square (R^2^) value of the linear regression was 0.9997.

**Fig. 2:**
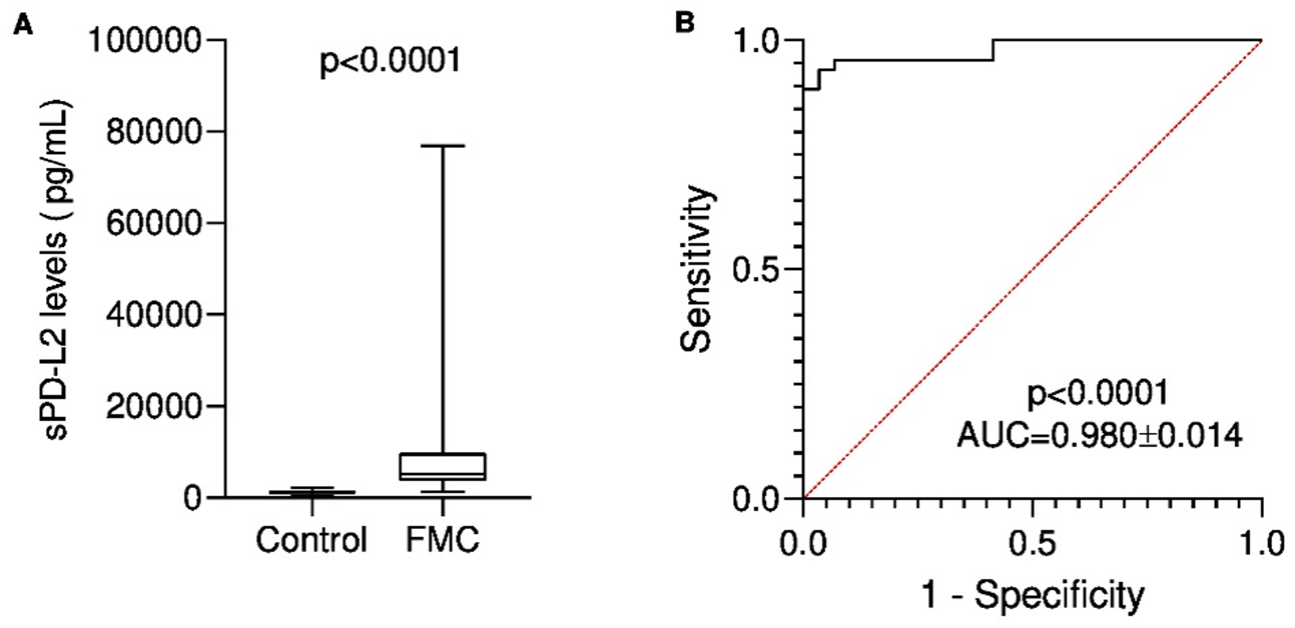
(**A**) Serum PD-L2 (sPD-L2) concentrations in Control and FMC groups. Data are shown as Tukey box-and-whisker plots with individual values. Cats with mammary carcinoma (FMC group) had higher serum PD-L2 levels than healthy cats (Control group). Statistical significance was assessed using the Mann-Whitney U test; *p* < 0.0001. (**B**) Receiver-operating characteristic (ROC) curve evaluating the diagnostic performance of the PD-L2 ELISA assay to differentiate cats with mammary carcinoma from healthy controls. The area under the curve (AUC) was 0.980 ± 0.014 (95% CI: 0.9529 to 1.000, *p* < 0.0001). The optimal *cut-off* value of 1934 pg/mL, determined using the Youden index, yielded a sensitivity of 93.6% and a specificity of 96.6%.

Serum PD-L2 concentrations were calculated from a standard linear regression using serial dilutions of recombinant PD-L2 with known concentrations (0.0005x – 0.0623, R^2^ = 0.9997 for rPD-L2, Fig. 1). Cats with mammary carcinoma (FMC group) showed elevated serum PD-L2 levels (median = 5283 pg/mL; mean = 21950 pg/mL; range of values: 1398-536925 pg/mL) compared with controls (median = 1274 pg/mL; mean = 1271 pg/mL; range of values: 412–2189 pg/mL), with a p value < 0.0001 (Fig. 2A). ROC analysis identified an optimal cutoff of 1934 pg/mL to distinguish diseased from healthy cats, with a specificity of 96.6%, a sensitivity of 93.6% and AUC of 0.980 (Fig. 2B).

### Cats with HER2-positive and triple-negative (TN) mammary carcinomas showed higher serum PD-L2 levels than those with other molecular subtypes

**Fig. 3:**
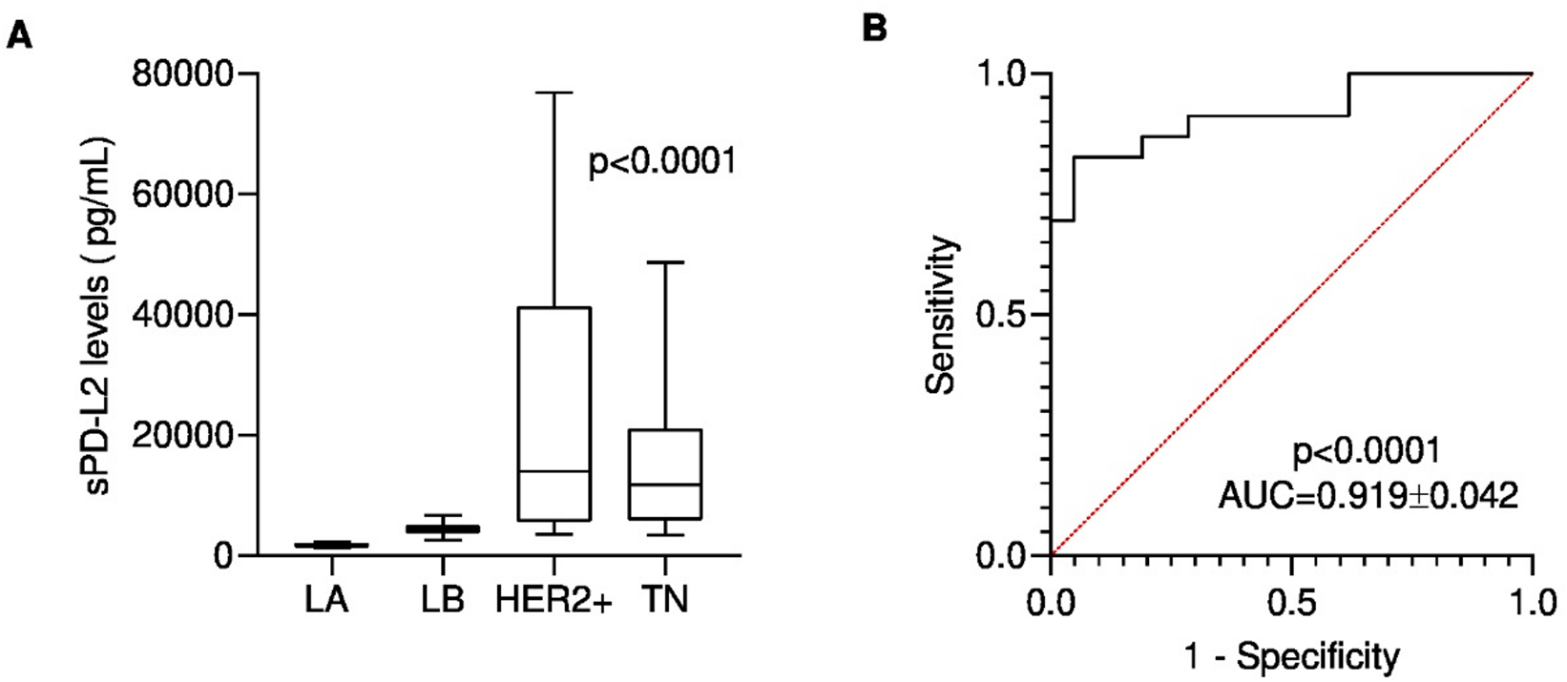
(**A**) Serum PD-L2 (sPD-L2) concentrations among the different mammary carcinoma subtypes. Mammary carcinomas were classified accordingly to the St. Gallen International Expert Consensus panel guidelines. To compare circulating PD-L2 levels between animals with different molecular feline mammary carcinoma subtypes (LA - Luminal A; LB - Luminal B; HER2+- LB-HER2 + and HER2+; TN normal-like and basal-like Triple Negative). Cats with HER2-overexpressing and TN tumors had higher serum PD-L2 levels. Data are shown as Tukey box-and-whisker plots with individual values. Statistical significance was assessed using Kruskal-Wallis’s test; *p* < 0.0001. (**B**) Receiver-operating characteristic (ROC) curve evaluating the diagnostic performance of the PD-L2 ELISA assay to differentiate the most aggressive (HER2-positive and TN) from less aggressive (LA and LB) mammary carcinomas. The area under the curve (AUC) was 0.919 ± 0.042 (95% CI: 0.8366 to 1.000, *p* < 0.0001). The optimal *cut-off* value of 5499 pg/mL, determined using the Youden index, yielded a sensitivity of 82.6% and a specificity of 95.2%.

When diseased cats were grouped by molecular subtype, HER2-positive and TN tumors showed significantly elevated serum PD-L2 levels compared to Luminal A or Luminal B tumors (*p* < 0.0001, Fig. 3A). Pairwise comparisons using the Kruskal–Wallis’s test followed by Dunn’s multiple comparisons test revealed significant differences between HER2-positive and Luminal A (*p* = 0.0001) and between HER2-positive and Luminal B tumors (*p* = 0.030), as well as between TN and Luminal A (*p* = 0.0001) and between TN and Luminal B tumors (*p* = 0.026). No significant differences were observed between HER2-positive and TN tumors (*p* > 0.9999) or between Luminal A and Luminal B tumors (*p* = 0.18).

ROC analysis determined 5499 pg/mL as the optimal cut-off value to differentiate cats with HER2-positive and TN subtypes from cats with the other subtypes, with a specificity of 95.2%, sensitivity of 82.6% and an AUC of 0.919 (Fig. 3B).

## Serum PD-L2, PD-L1 and PD-1 levels are positively correlated

**Fig. 4:**
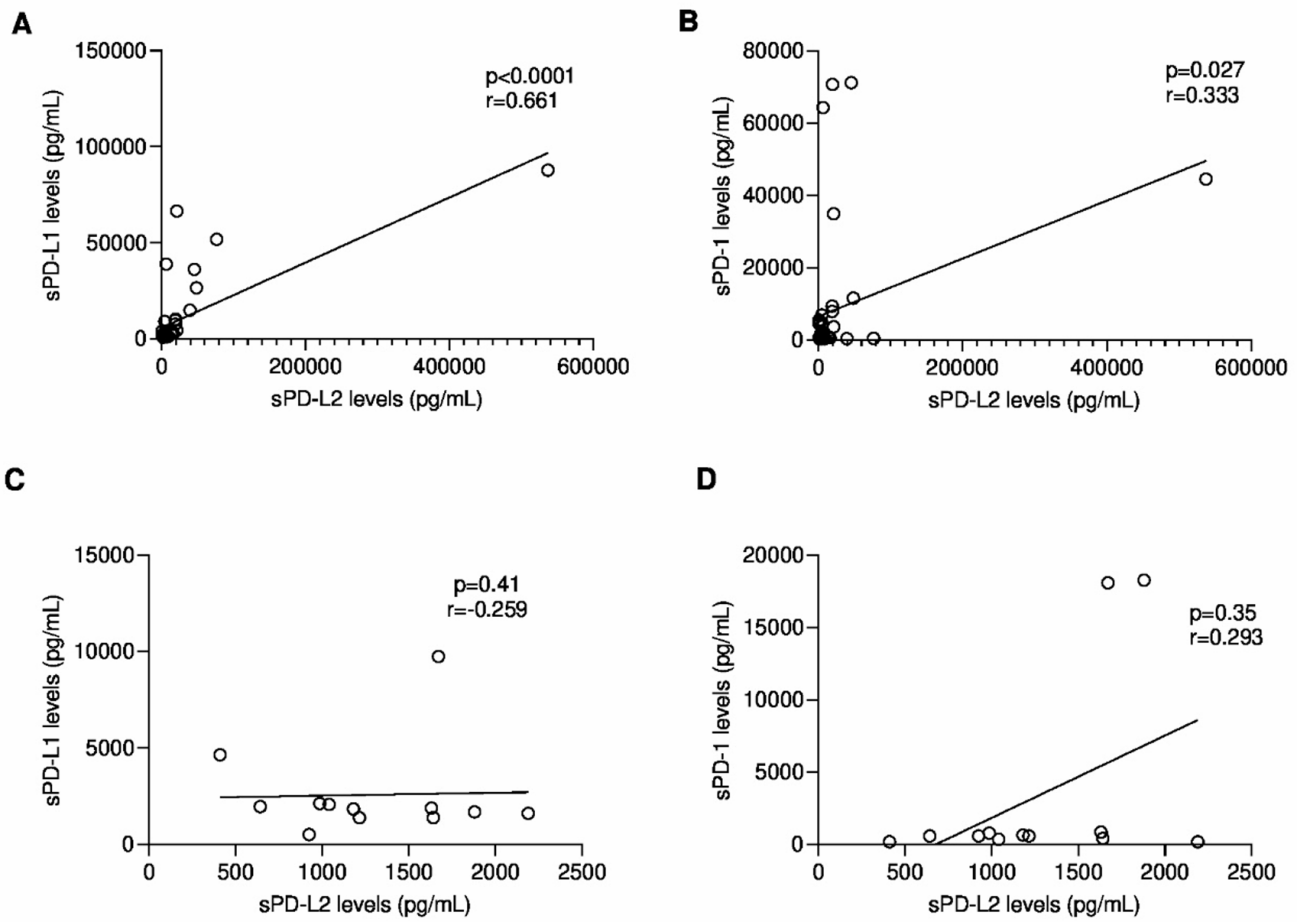
Spearman Coefficient Correlation analysis between serum PD-L2 levels and (**A**) serum PD-L1 and (**B**) serum PD-1 in FMC group (**C**) serum PD-L1 and (**D**) serum PD-1 in Control group. Each point represents one animal. The correlation value of PD-L2 and PD-L1 is strong (*r* = 0.661, *p* < 0.0001; 95% CI: 0.4457 to 0.8039), while with PD-1 it is weak (*r* = 0.333, *p* = 0.027; 95% CI: 0.0313 to 0.5794) in cats with mammary carcinoma. No correlation was observed between serum PD-L2 and serum PD-L1 (*r*= -0.259; *p* = 0.41; 95% CI: -0.7342 to 0.3863) and serum PD-1 (*r* = 0.293; *p* = 0.35; 95% CI: -0.3549 to 0.7506) in heathy cats.

The Spearman correlation analysis revealed positive associations between serum PD-L2, PD-L1 and PD-1 levels in cats with mammary carcinoma. A strong correlation was observed between sPD-L2 and sPD-L1 levels (*r* = 0.661, *p* < 0.0001, 95% CI: 0.4457 to 0.8039; Fig. 4A), while a moderate association was found between sPD-L2 and sPD-1 (*r* = 0.333, *p* = 0.027, 95% CI: 0.03126 to 0.5794, Fig. 4B). In healthy cats correlations were week for both pairs: sPD-L2 and sPD-L1 (*r*=-0.259; *p* = 0.41; 95% CI: -0.7342 to 0.3863; Fig. 4C), and between sPD-L2 and sPD-1 levels (*r* = 0.293; *p* = 0.35; 95% CI: -0.3549 to 0.7506; Fig. 4D).

**Fig. 5:**
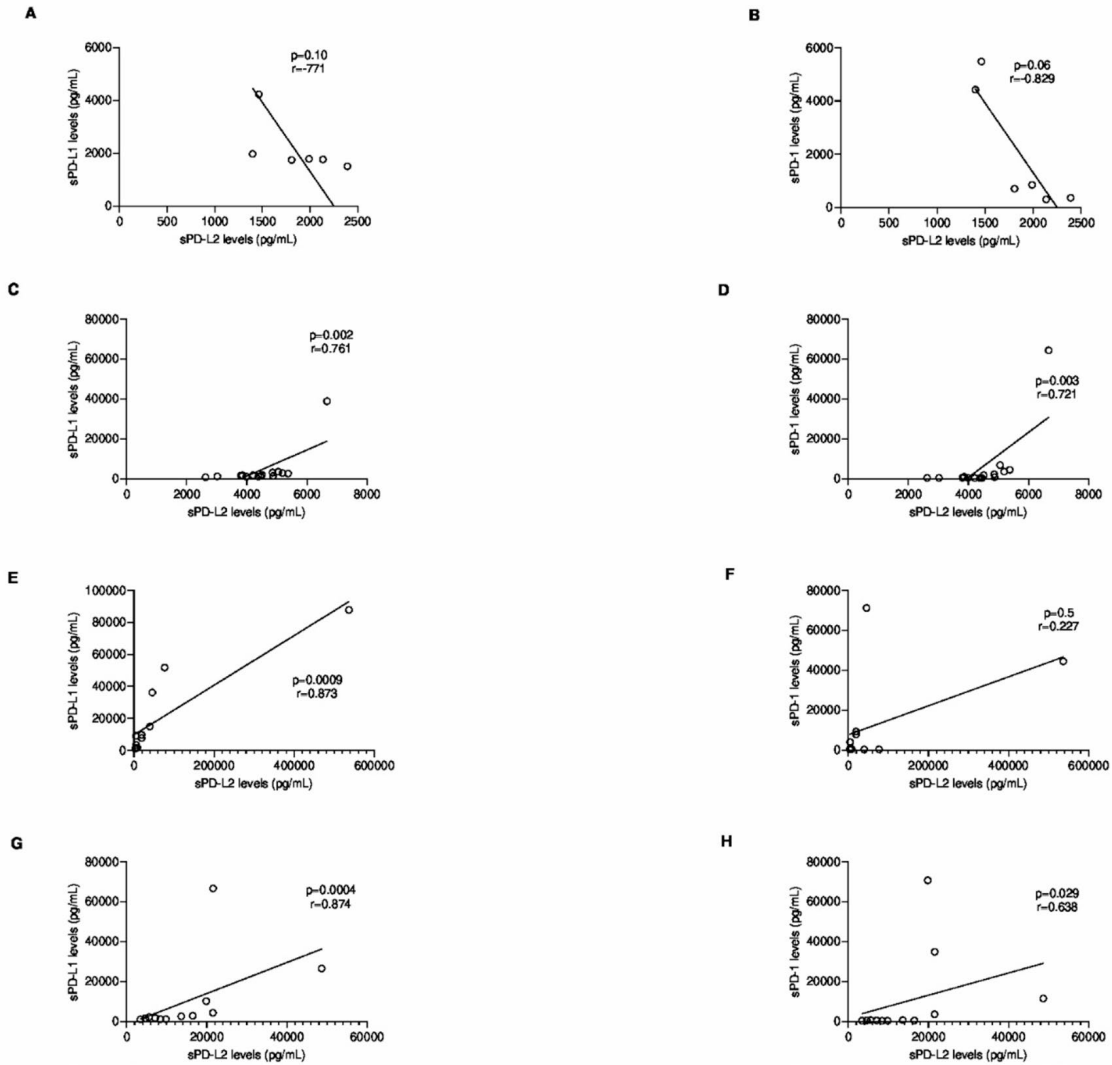
Spearman correlation analysis between serum PD-L2 levels and serum PD-L1 and serum PD-1 in cats with Luminal A (LA), Luminal B (LB), HER2-positive and Triple Negative (TN) mammary carcinoma subtypes, respectively. Each point represents one animal. No correlation was observed between sPD-L2 levels and (**A**) sPD-L1 (*r*= -0.771; *p* = 0.10) and (**B**) PD-1 (*r*= -0.829; *p* = 0.06) in animals with Luminal A (LA) carcinomas. A strong positive correlation between serum PD-L2 and (**C**) PD-L1 (*r* = 0.761, *p* = 0.002, 95% CI: 0.3930 to 0.9187) and (**D**) PD-1 (*r* = 0.721, *p* = 0.003, 95% CI: 0.3159 to 0.9037) levels in cats with Luminal B (LB) mammary carcinoma subtype was observed. In HER2-positive (HER2+) cats, a very strong positive correlation between sPD-L2 and (**E**) sPD-L1 (*r* = 0.873, *p* = 0.0009, 95% CI: 0.5587 to 0.9679) was shown, while no correlation between serum PD-L2 and (**F**) PD-1 was verified(*r* = 0.227; *p* = 0.5; 95% CI: -0.4479 to 0.7374). In animals with Triple Negative (TN) carcinomas, a very strong positive correlation was observed between serum PD-L2 levels and (**G**) PD-L1 levels (*r* = 0.874; *p* = 0.0004; 95% CI: 0.5900 to 0.9656) and a strong positive correlation between serum PD-L2 and (H) PD-1 levels (*r* = 0.638; *p* = 0.029; 95% CI: 0.0811 to 0.8910).

When cats were grouped according to their tumor molecular subtype, Luminal A tumors exhibited strong negative correlations between sPD-L2 and sPD-L1 levels (*r*=-0.771; *p* = 0.10; Fig. 5A), and between sPD-L2 and sPD-1 levels (*r*=-0.829; *p* = 0.06; Fig. 5B). In contrast, Luminal B tumors showed a significant and strong positive correlations for both pairs: sPD-L2 and sPD-L1 levels (*r* = 0.761, *p* = 0.0015, 95% CI: 0.3930 to 0.9187, Fig. 5C) and sPD-L2 and sPD-1 levels, (*r* = 0.721; *p* = 0.003; 95% CI: 0.3159 to 0.9037; Fig. 5D).

In parallel, animals with HER2-overexpressing tumors showed a very strong positive correlation between sPD-L2 and sPD-L1 levels (*r* = 0.873; *p* = 0.0009; 95% CI: 0.5587 to 0.9679; Fig. 5E), whereas a weak correlation was found between sPD-L2 and sPD-1 levels (*r* = 0.227; *p* = 0.5; 95% CI: -0.4479 to 0.7374; Fig. 5F). Finally, cats with TN mammary carcinomas exhibited strong positive correlations for both pairs: sPD-L2 and sPD-L1 levels (*r* = 0.874; *p* = 0.0004; 95% CI: 0.5900 to 0.9656; Fig. 5G), and sPD-L2 and sPD-1 levels (*r* = 0.638; *p* = 0.029; 95% CI: 0.08110 to 0.8910; Fig. 5H).

### Serum PD-L2 levels are positively correlated with other immune checkpoint molecules

**Fig. 6:**
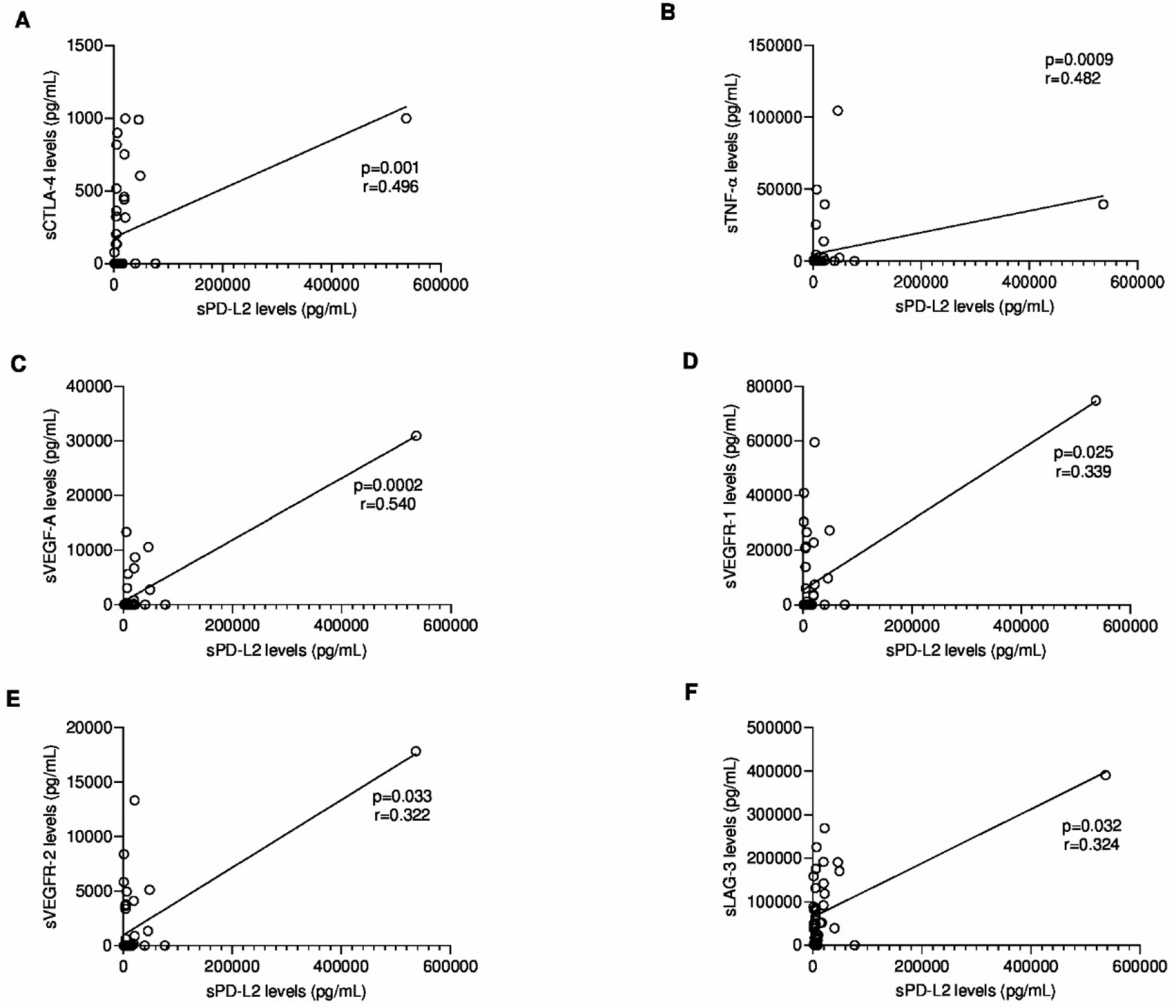
Spearman correlation analysis between serum PD-L2 levels and serum (**A**) CTLA-4, (**B**) TNF-α, (**C**) VEGF-A, (**D**) VEGFR-1, (**E**) VEGFR-2 and (**F**) LAG-3 levels in FMC group. Each point represents one animal. A moderate correlation was observed between sPD-L2 and sCTLA-4 (*r* = 0.496, *p* = 0.001, 95% CI: 0.2135 to 0.7021), sTNF-α (*r* = 0.482, *p* = 0.0009, 95% CI: 0.2077 to 0.6863) and sVEGF-A (*r* = 0.540, *p* = 0.0002, 95% CI: 0.2806 to 0.7252). While a weak correlation was revealed between sPD-L2 and sVEGFR-1 (*r* = 0.339, *p* = 0.025, 95% CI: 0.03752 to 0.5835), sVEGFR-2 (*r* = 0.322, *p* = 0.033, 95% CI: 0.01868 to 0.5710) and sLAG-3 (*r* = 0.324, *p* = 0.032, 95% CI: 0.02065 to 0.5723).

Moderate positive correlations were found between sPD-L2 and sCTLA-4 levels (*r* = 0.496, *p* = 0.001, 95% CI: 0.2135 to 0.7021; Fig. 6A), sTNF-α levels (*r* = 0.482, *p* = 0.0009, 95% CI: 0.2077 to 0.6863; Fig. 6B) and sVEGF-A levels (*r* = 0.540, *p* = 0.0002, 95% CI: 0.2806 to 0.7252; Fig. 6C). Moreover, a weak positive correlation was observed between sPD-L2 and sVEGFR-1 levels (*r* = 0.339; *p* = 0.025; 95% CI: 0.03752 to 0.5835; Fig. 6D), sVEGFR-2 levels (*r* = 0.322; *p* = 0.033; 95% CI: 0.01868 to 0.5710; Fig. 6E) and sLAG-3 levels (*r* = 0.324; *p* = 0.032; 95% CI: 0.02065 to 0.5723; Fig. 6F).

In contrast, no significant correlations were detected between sPD-L2 and either sCTLA-4 levels (*r* ≈ 0), sTNF-α levels (*r* = 0.521; *p* = 0.091; 95% CI: -0.09405 to 0.8485), sVEGF-A levels (*r* = 0.306; *p* = 0.5; 95% CI: -0.3424 to 0.7567), sVEGFR-1 levels (*r*=-0.118; *p* = 0.71; 95% CI: -0.6592 to 0.5034), sVEGFR-2 levels (*r*=-0.118; *p* = 0.71; 95% CI: -0.6592 to 0.5034) and sLAG-3 levels (*r* = 0.007; *p* = 0.99; 95% CI: -0.5818 to 0.5916).

### Serum PD-L2 levels are correlated with PR and HER2 expression and the Ki-67 index

**Fig. 7:**
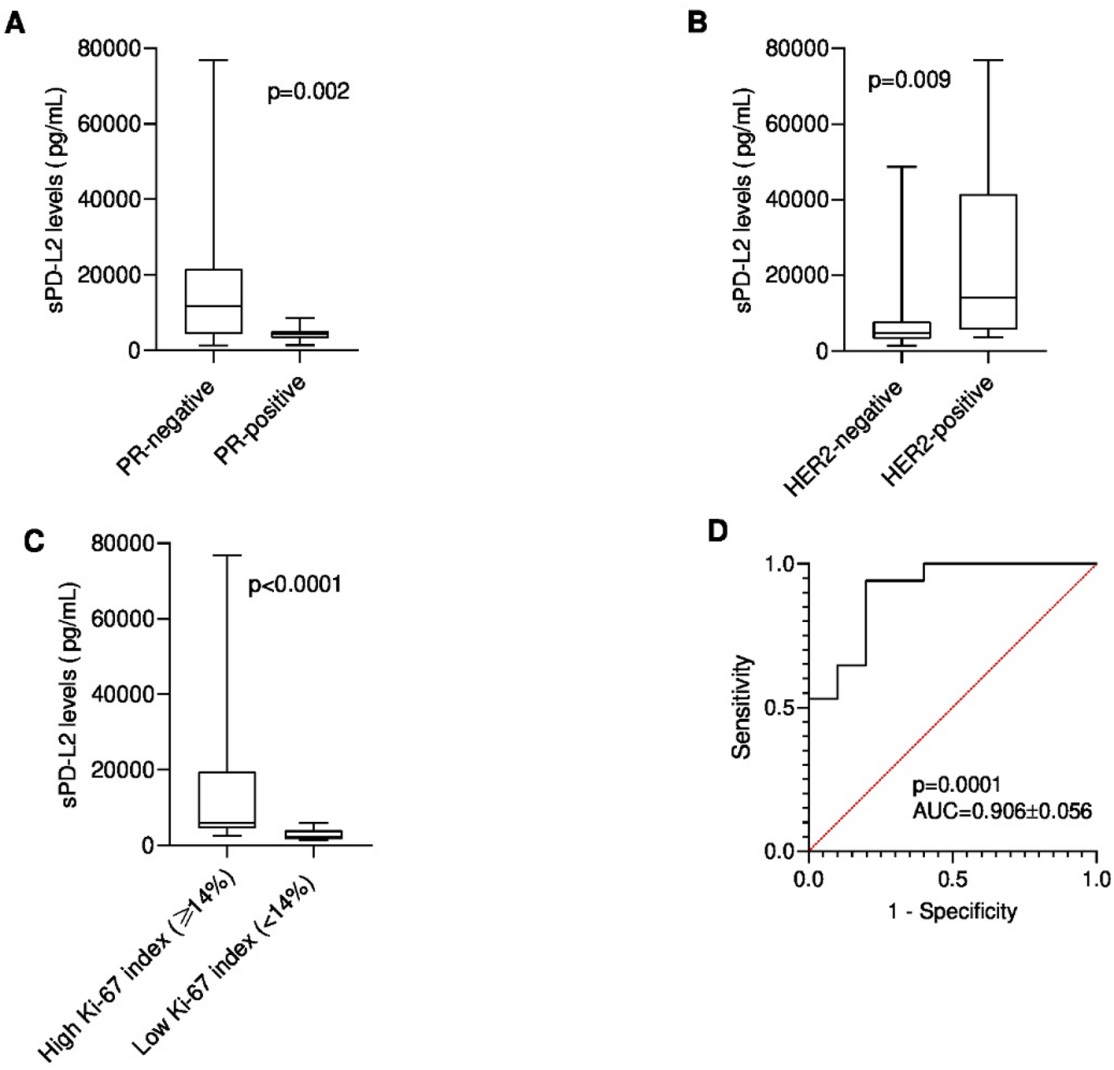
Serum PD-L2 (sPD-L2) concentrations considering (**A**) Progesterone Hormone (PR) Status, (**B**) Human epidermal growth factor receptor 2 (HER2) Status and Ki-67 index levels (**C**) in FMC group. Data are shown as Tukey box-and-whisker plots with individual values. Statistical significance was assessed using the Mann-Whitney U test. PR-positive cats have a negative correlation with PD-L2 serum levels, *p* = 0.002. HER2-positive cats have a positive correlation with PD-L2 serum levels, *p* = 0.009. High Ki-67 index (≥ 14%) are associated with higher serum PD-L2 levels, *p* < 0.0001. (**D**) Receiver-operating characteristic (ROC) curve of serum PD-L2 levels of cats with mammary carcinoma (FMC) and Ki-67 levels. The best serum PD-L2 *cut-off* value to differentiate disease with elevated Ki-67 index (≥ 14%) from low Ki-67 index (< 14%) (3732 pg/mL) was determined to maximize the sum of the sensitivity (94.1%) and specificity (80.0%) using the Youden index (sensitivity + specificity − 1). The estimated AUC was 0.906 ± 0.056 (95% CI: 0.7955 to 1.000, *p* = 0.0001).

In cats with mammary carcinoma, sPD-L2 levels were significantly associated with PR status (*p* = 0.002, Fig. 7A), HER2 status (*p* = 0.009, Fig. 7B) and the Ki-67 index (*p* < 0.0001, Fig. 7C). Indeed, results revealed that cats with PR-positive mammary carcinomas showed lower sPD-L2 levels than those with PR-negative tumors (Fig. 7A), whereas HER2-overexpressing tumors and Ki-67 indices ≥ 14% were linked to higher sPD-L2 concentrations (Fig. 7B and C, respectably). Additionally, the ROC analysis defined 3732 pg/mL as the optimal cut-off value to distinguish tumors with Ki-67 indices ≥ 14% (specificity: 80.0%; sensitivity: 94.1%; AUC = 0.906, Fig. 7D).

No significant associations were found between serum PD-L2 and the different clinicopathological features, namely, age (*p* = 0.51), breed (*p* = 0.69), reproductive status (*p* = 0.21), contraceptive (*p* = 0.71), treatment (*p* = 0.12), number (*p* = 0.37), location (*p* = 0.90) or size of tumor lesions (*p* = 0.16), histopathological classification (*p* = 0.23), malignancy grade (*p* = 0.73), tumor necrosis (*p* = 0.39), metastases (*p* = 0.43), lymphatic invasion (*p* = 0.13), lymphocytic infiltration (*p* = 0.45), cutaneous ulceration (*p* = 0.17), lymph node involvement (*p* = 0.95) or TNM stage (*p* = 0.20). At the molecular level, no significant associations were identified between serum PD-L2 and ER and CK5/6 status (*p* = 0.078, *p* = 0.58, respectively).

### Cats with TN mammary carcinomas had the poorest DFS and OS

**Fig. 8:**
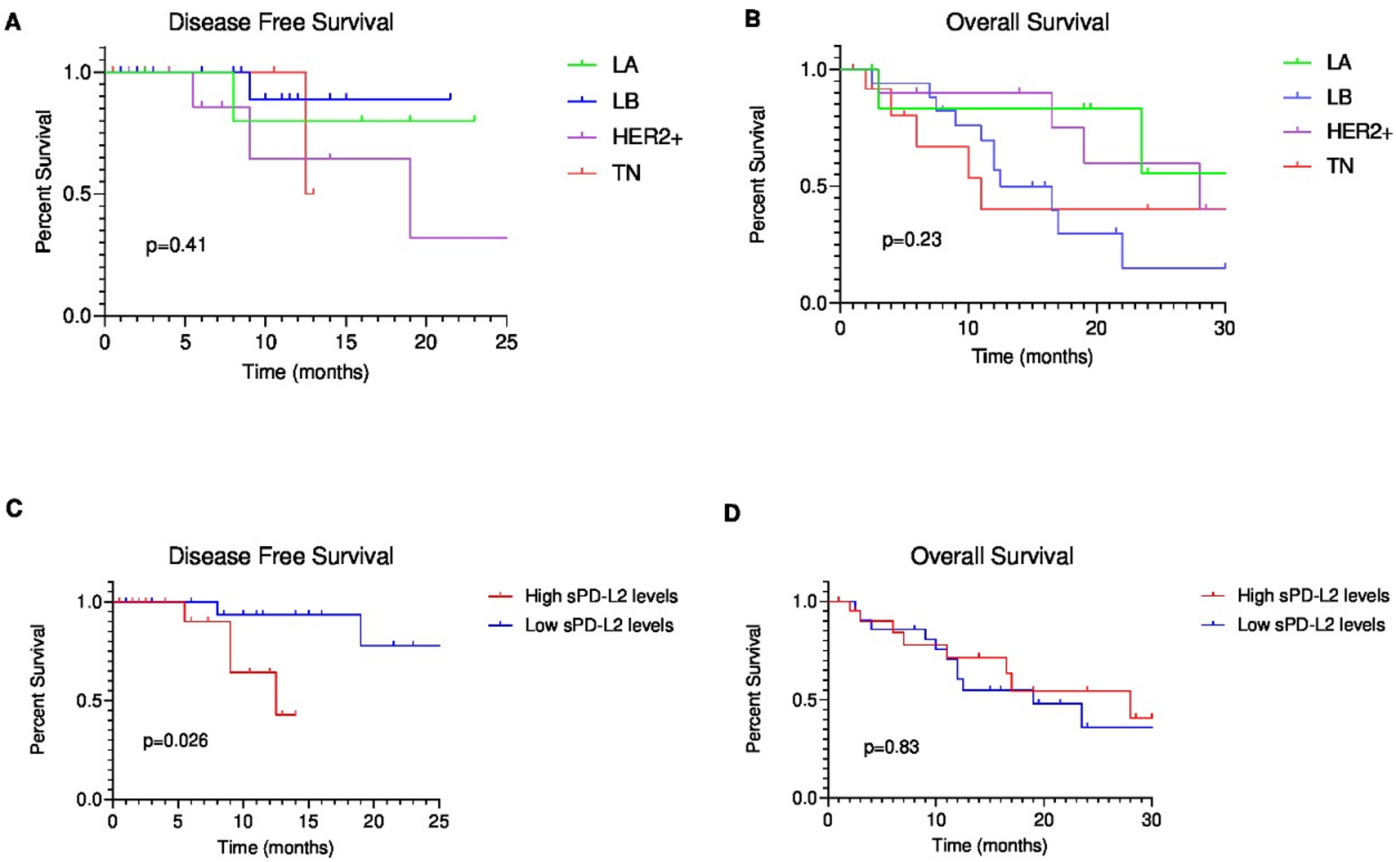
Kaplan–Meier method (log-rank test) for DFS and OS analysis in FMC group, namely Luminal A (LA), Luminal B (LB), HER2-positive (HER2+) and Triple Negative (TN). No statistical association was found between the different molecular mammary carcinoma subtypes and (**A**) DFS (*p* = 0.41) and (**B**) OS (*p* = 0.23). While a statistical association was verified between high and low sPD-L2 levels based on median values and (**C**) DFS (*p* = 0.026). However, no correlation was observed between high and low sPD-L2 levels based on median values and (**D**) OS (*p* = 0.83).

DFS was shortest in cats with TN tumors (median = 2.0 months; *n* = 11), followed by HER2-positive tumors (median = 6.65 months; *n* = 10), luminal B tumors (median = 9.5 months; *n* = 16) and luminal A tumors (median = 17.5 months; *n* = 6) (Fig. 8A). OS showed the same pattern: TN tumors had the lowest OS (median = 5.0 months; *n* = 13) compared with luminal B (median = 12.0 months; *n* = 17), HER2-positive (median = 17.75 months; *n* = 10), and luminal A tumors (median = 19.5 months; *n* = 7; Fig. 8B). Despite these trends, no statistically significant differences in DFS or OS were detected among molecular subtypes (*p* = 0.41, Fig. 8A; *p* = 0.23, Figs. 8B, respectively). Median 95% confidence intervals could not be reliably computed because the underlying Kaplan–Meier estimates/individual time-to-event data required for CI estimation were not available.

When cats were grouped by sPD-L2 levels relative to the median, DFS differed significantly (*p* = 0.026, *n* = 40, Fig. 8C), with higher sPD-L2 associated with shorter DFS. No association was observed for OS (*p* = 0.83, *n* = 43, Fig. 8D).

## Discussion

In humans, the PD-1/PD-L1/PD-L2 axis promotes T-cell apoptosis and anergy, supporting tumor growth^56^, being associated with poor prognosis^57^. Recently, the PD-L2 expression by tumor cells has gained further attention^15,27^ as it’s overexpression influences the effectiveness of anti-PD-L1/PD-1 therapies^15^ and promotes tumor progression^15,27,28^. Although the prognostic significance of PD-L2 expression varies across cancer types^58–60^, elevated serum PD-L2 levels has been linked with immunotherapy resistance in non-small cell lung cancer^20^, advanced epithelial ovarian carcinoma^61^ and breast cancer^32,62^, consistent with its role in immune escape and tumor development^63^.

Taking into consideration the biological relevance of PD-L2 and the similarities between FMC and human breast cancer^64,65^, quantification of serum PD-L2 may offer a rapid and non-invasive diagnostic tool, while PD-L2-targeted therapies may improve treatment outcomes^44^. Indeed, a monoclonal antibody (1A1-2) recognizing canine and feline PD-1 was recently developed^66^, while a novel anti-feline PD-L1 monoclonal antibody (CL1Mab-7) enables selection of candidates for PD-L1-based immunotherapy^44^. Interestingly, a monoclonal antibody anti-PD-L1 is also available for canine malignant oral melanoma and undifferentiated sarcoma^67,68^.

In our study cats with mammary carcinoma exhibited significantly higher serum PD-L2 levels than healthy controls (*p* < 0.0001), mirroring findings reported in HBC patients^32,69^. ROC analysis identified 1934 pg/mL as the optimal diagnostic sPD-L2 cut-off value to differentiate both groups, further suggesting its potential use as a diagnostic biomarker. Consistent with reports in humans^69^, cats with HER2-positive and TN mammary carcinomas showed the highest sPD-L2 levels (*p* < 0.0001). Given that these subtypes are among the most aggressive in this species^39^, our findings suggest that sPD-L2 may represent a putative prognostic biomarker, with 5499 pg/mL identified as the optimal cut-off.

Our study further showed that sPD-L2, sPD-L1 and sPD-1 display broadly concordant patterns across tumor subtypes. Strong positive correlations were observed between sPD-L2 and sPD-L1 in HER2-positive, TN or LB tumors (*r* = 0.873, *p* = 0.0009; *r* = 0.874, *p* = 0.0004; *r* = 0.761, *p* = 0.002, respectively). Strong correlations were also found with sPD-1 in TN and LB tumors (*r* = 0.638, *p* = 0.029; *r* = 0.721, *p* = 0.003; respectively). Taken together, these findings are consisted with T-cell suppression^70–72^. PD-1 and PD-L1 are established prognostic markers in several solid tumors, including aggressive renal-cell carcinoma^73^, hepatocellular carcinoma^74^, non-small-cell lung cancer^75^, and oral cancer^76^ and have also been implicated in feline mammary carcinoma^43^. Mechanistically, elevated soluble PD-L1 can inhibit T-cell activation^77^, and similar immunomodulatory effects may be expected from higher sPD-L2 levels. Moreover, the genomic proximity of PD-L1 and PD-L2 and their reported co-expression patterns provide a plausible biological basis for the strong correlations observed. These findings extend our previous report of elevated sPD-1 and sPD-L1 in HER2-positive and TN FMC^43^, in line with observations in HBC^30,31^, and further emphasizing a conserved role of the PD-1/PD-L1/PD-L2 axis across species^44^.

Notably, no correlation between sPD-L2 and sPD-L1 was found in Luminal A tumors, underscoring the need to quantify both ligands when selecting candidates for PD-1/PD-L1/PD-L2-directed therapies, as recommended for HBC patients^8^.

Because tumor progression significantly depends on immune checkpoint concentrations^78,79^, the positive correlations between sPD-L2 and sCTLA-4 (*r* = 0.496, *p* = 0.001), sTNF-α (*r* = 0.482, *p* = 0.0009), sVEGF-A (*r* = 0.540, *p* = 0.0002), sVEGFR-1 (*r* = 0.339, *p* = 0.025), sVEGFR-2 (*r* = 0.322, *p* = 0.033) and sLAG-3 (*r* = 0.324, *p* = 0.032), may indicate a stable and persistent immunosuppression status in FMC. In HBC patients, CTLA-4 competes with CD28 for CD80 and CD86 ligands, interfering with T cell activation^80,81^, regulates IL-2 production, naive CD4 + T cell differentiation, T cell effector functions, B cell responses by T follicular helper cells and modulates T follicular regulatory cell functions^82^. In FMC, CTLA-4 overexpression correlates with FOXP3 + TILs expression and immune tolerance^83^. In parallel, TNF-α overexpression correlates with tumor proliferation, high malignancy, metastasis, a poor prognosis^84^, and is involved in tumor cell survival and PD-L1/PD-L2 stabilization in TNBC^85,86^, consistent with feline data^43^. VEGF-A, secreted by cancer and stromal cells^87,88^, promotes angiogenesis via VEGFR-1 and VEGFR-2 activation in FMC^89^; in humans its associated with HER2-positive and TN tumors^90,91^, poor prognosis and reduced DFS/OS^92^, findings parallel in FMC^89^. LAG-3, associated with T cell dysfunction and aggressive clinicopathological features^93^, correlates positively with PD-1 expression on CD4 + and/or CD8 + TILs in TME^38^; serum LAG-3 also correlates with TIM-3 in FMC^65^.

In this study, no significant associations were found between sPD-L2 levels and clinicopathological features such as age (*p* = 0.51), breed (*p* = 0.69), reproductive or contraceptive status (*p* = 0.21 and *p* = 0.71, respectively), treatment (*p* = 0.12), tumor number (*p* = 0.37), location (*p* = 0.90) or size (*p* = 0.16), histopathological classification (*p* = 0.23), malignancy grade (*p* = 0.73), necrosis (*p* = 0.39) metastasis (*p* = 0.43), lymphatic invasion (*p* = 0.13), lymphocytic infiltration (*p* = 0.45), cutaneous ulceration (*p* = 0.17), regional lymph node involvement (*p* = 0.95) or TMN stage (*p* = 0.20), partially aligning with HBC data^32^. Given that histopathological subtype showed no association with sPD-L2, in contrast to molecular subtype, adoption of FMC immunophenotyping^39^ is strongly advised in veterinary practice. This overall lack of correlations suggests that circulating sPD-L2 may not chiefly reflect tumor morphology or disease extent at diagnosis, but rather other aspects of tumor biology and host–tumor immune crosstalk. Similar observations have been reported in human breast cancer, where soluble immune-checkpoint markers often show limited associations with conventional clinicopathological parameters^32^. The absence of associations with histological subtype and grade may also stem from heterogeneity within these categories and interobserver variability. Notably, the discrepancy between histopathological findings and molecular subtype–related differences support the view that immunophenotyping captures biologically relevant variation not evident on routine histology. Clinically, this underscores the value of incorporating ER/PR/HER2-based classification in feline mammary carcinoma to refine prognostic stratification and interpretation of immune biomarkers^51^. However, non-significant results do not preclude modest effects, and limited sample size may have reduced power; larger prospective studies with multivariable analyses are warranted.

At the molecular level, sPD-L2 levels were not significantly associated with ER status (*p* = 0.078). Independently, ER-positive tumors have been associated with lower mortality in breast cancer^32,94,95^ and in feline mammary carcinoma^51^, whereas higher sPD-L2 concentrations in our study were observed mainly in more aggressive molecular subtypes. A significant association was found with PR status (*p* = 0.002). Notably, PR negativity is common in malignant feline mammary tumors^96^, and in our study higher sPD-L2 levels were also observed in HER2-positive and triple-negative carcinomas. As in HBC^32,97^, sPD-L2 levels were positively correlated with a HER2-positive status (*p* = 0.009), which might reflect a potential diagnostic and prognostic relevance, warning further investigation.

Feline mammary carcinomas with Ki-67 indices ≥ 14% had significantly higher sPD-L2 levels (*p* < 0.0001), and 3732 pg/mL was identified as the optimal cut-off to discriminating high- from low-proliferating tumors. These findings are consistent with reports on human breast cancer^32^ and may reflect adverse biological behavior in feline mammary carcinoma^51^.

Finally, our data shows that sPD-L2 levels were significantly associated with DFS based on median values (*p* = 0.026), but not with OS (*p* = 0.83). The observed association between higher sPD-L2 concentrations and shorter DFS is consistent with a potential role of the PD-1/PD-L2 axis in immune evasion and earlier recurrence/progression in feline mammary carcinoma, suggesting that sPD-L2 may serve as a surrogate marker of residual microscopic disease or of a more aggressive tumor–host immune interaction. Conversely, the absence of a significant association with OS may reflect the multifactorial determinants of survival in this population, including heterogeneity in disease stage and tumor biology, as well as limited statistical power to detect OS differences in a study of this size. From a clinical standpoint, these findings suggest that sPD-L2 may be more informative for estimating relapse risk and timing than for predicting OS, and they support further validation of sPD-L2 as a prognostic biomarker and as a potential tool for postoperative risk stratification in larger, prospectively followed cohorts.

The present study has several limitations that should be acknowledged. First, the ELISA kit employed is approved for use with human blood samples and has not yet been validated for feline specimens. Second, sample sizes differed between the control and diseased groups, and, within the diseased cohort, the various molecular subtypes of mammary carcinoma were not equally represented. Third, although the study is novel and provides a substantial amount of data, it relies on multiple statistical associations and does not include multivariate statistical analyses. Fourth, the serum bank samples varied with respect to their source, time of collection, and the personnel involved in sample processing. Accordingly, additional studies are needed to validate the present findings and to examine the relationship between serum PD-L2 concentrations and PD-L2 expression in tumor cells in cats with mammary carcinoma. This is potentially relevant, as PD-L2 overexpression has been reported as an indicator of poor prognosis in certain tumor types^32^. Moreover, the role of PD-L2 in oncogenesis remains incompletely understood, and only a limited number of studies have assessed PD-L2 in the plasma of human cancer patients, particularly in breast carcinoma. It may also be informative to compare serum PD-L2 levels and tissue expression between cats with mammary carcinoma and cats with benign mammary tumors or cystic lesions, in order to better define the clinical and prognostic value of circulating PD-L2 in feline mammary carcinoma. Finally, the limited access to complete clinical records—including missing dates of death—and the restricted assessment of recurrence at the attending veterinary clinics hindered consistent follow-up of the animals, whereas for the recent cases, recurrence and survival data will only become available at a later stage.

## Conclusion

Within the context of the present study, serum PD-L2 levels may have diagnostic and prognostic relevance in feline mammary carcinoma, particularly in the more aggressive molecular subtypes such as HER2-positive and TN. These observations are consistent with the findings reported in HBC patients, emphasizing the value of FMC as a comparative model. Finally, considering the exploratory scope of the present work and the aforementioned limitations, additional studies are required to elucidate the clinical relevance of PD-L2 in feline mammary carcinoma.

## Data Availability

The datasets used and/or analyzed during the current study are available from the corresponding author on reasonable request.
